# Supplementary material for: Carbon-Supported High-Loading Sub-4 nm PtCo Alloy Electrocatalysts for Superior Oxygen Reduction Reaction
Source: Nanomaterials (Basel). 2023 Aug 18;13(16):2367. doi: 10.3390/nano13162367 (PMC10458021; doi:10.3390/nano13162367)
Supplement: Supplementary file 1 [file nanomaterials-13-02367-s001.zip › nanomaterials-2453114-supplementary.pdf]

Supporting Information for

Carbon Supported High-Loading Sub-4 nm PtCo Alloy Electrocatalysts for Superior Oxygen Reduction Reaction

Linlin Xiang[a,b], Yunqin Hu[a], Yanyan Zhao[c], Sufeng Cao[d] and Long Kuai[a,b]*

[a]School of Chemical and Environmental Engineering, Anhui Laboratory of Clean Catalytic Engineering, Anhui Polytechnic University, Beijing Middle Road, Wuhu, 241000, China

[b]Institute of Energy, Hefei Comprehensive National Science Center, Anhui, Hefei, 230031, China

[c]The Rowland Institute at Harvard, 100 Edwin H Land Blvd, Cambridge, MA 02142, USA

[d]Aramco Boston Downstream Center, 400 Technology Square, Cambridge, MA, 02139, USA

*Corresponding author, Email: kuailong@ahpu.edu.cn

S1: Additional Figure and Figure captions

S2: Additional Tables

S1: Additional Figure and Figure captions

Figure S1

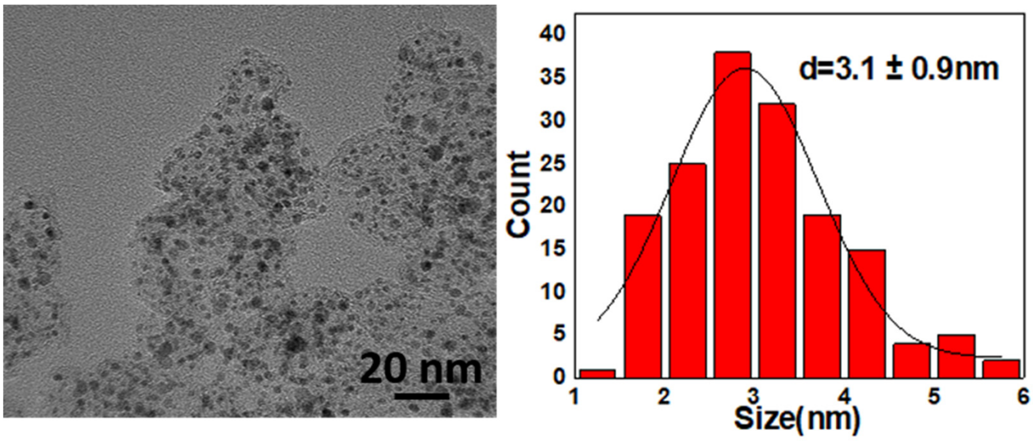

Figure S1. TEM image (left) and size distribution (right) of 40% Pt_3Cu_2/C catalyst.

Figure S2

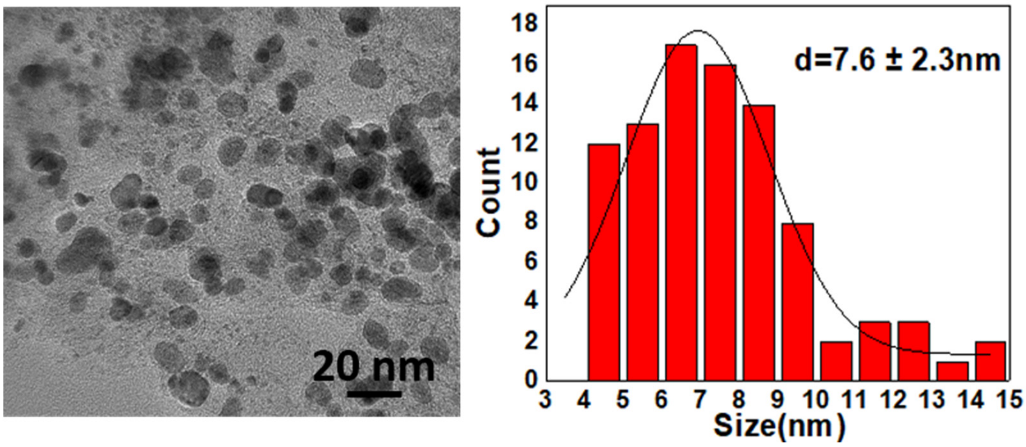

Figure S2. TEM image (left) and size distribution (right) of 40% Pt_3Fe_2/C catalyst.

Figure S3

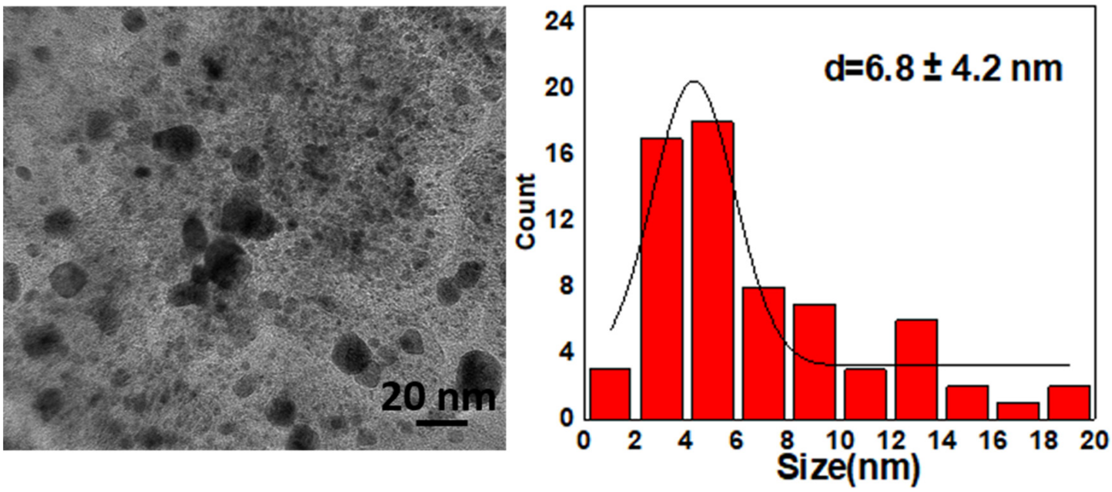

Figure S3. TEM image (left) and size distribution (right) of 40% Pt_3Ni_2/C catalyst.

Figure S4

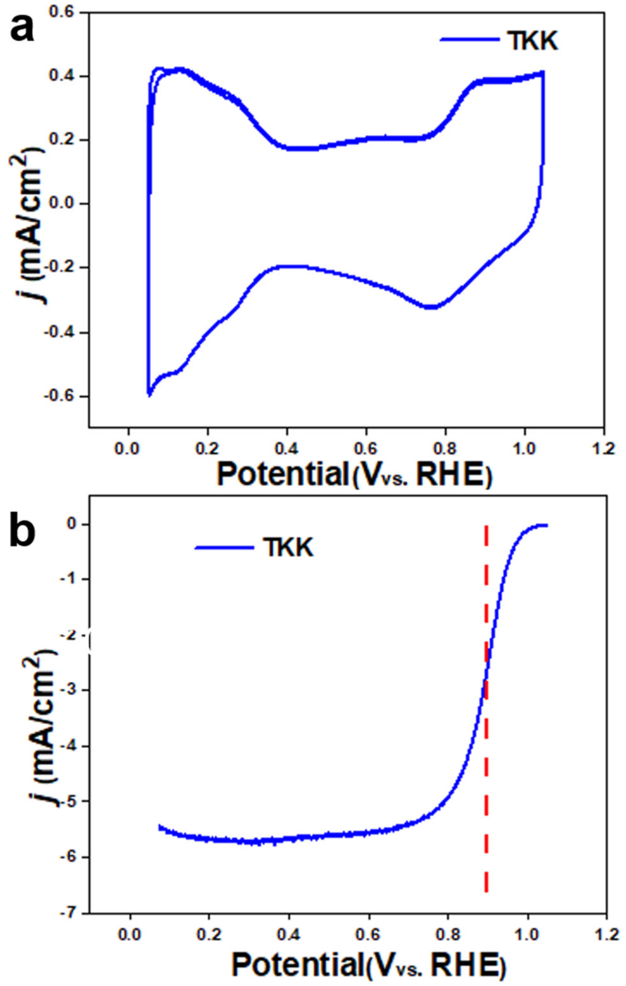

Figure S4. CV curve in N_2-saturated 0.1 M $HClO_4$ (a), iR-corrected LSV curve in O_2-saturated 0.1 M $HClO_4$ (b) of commercial PtCo/C catalysts (TANAKA TKK).

Figure S5

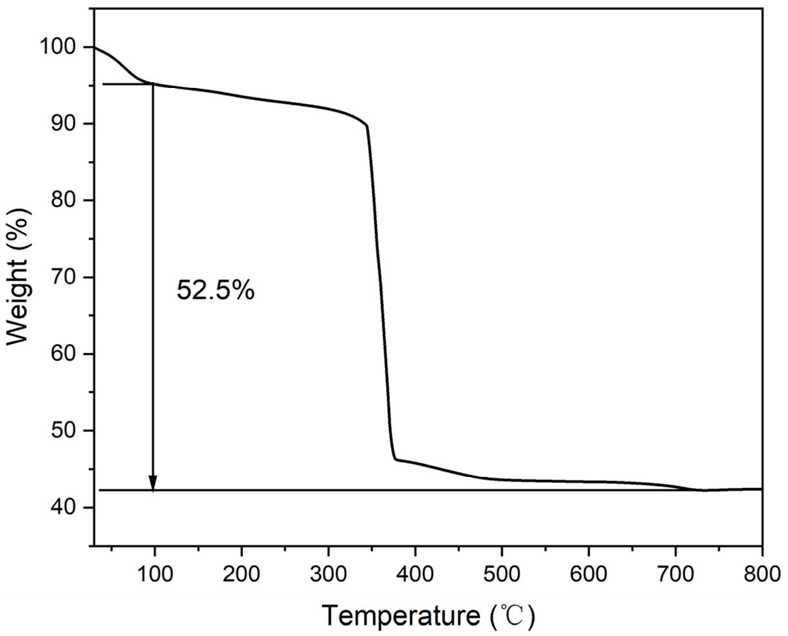

Figure S5. Thermogravimetric analysis (TGA) curves under the O_2 atmosphere flow from 30 °C to 800 °C of Pt_3Co_2/C-4 h.

Figure S6

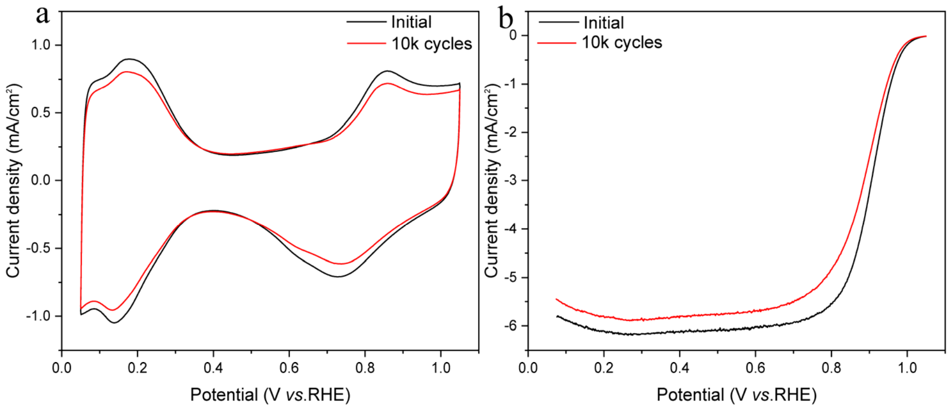

Figure S6. The CV curves in N_2-saturated 0.1 M $HClO_4$ (a) and iR-corrected LSV curves in O_2-saturated 0.1 M $HClO_4$ (b) of PtCo/C-4h before (black curves) and after (red curves) 10000 cycles.

S2: Additional Tables

Table S1. The calcination time, grain size, average particle size and ordering degree of Pt_3Co_2-4h, Pt_3Co_2-8h and Pt_3Co_2-12h catalysts were summarized respectively.

Catalyst	Calcination program	XRD size(nm)	STEM size(nm)	Alloying Degree(%)
Pt_3Co_2	560℃/ 4 h	2.7	2.8±0.8	73.6
Pt_3Co_2	560℃/ 8 h	3.8	3.5±1.0	78.7
Pt_3Co_2	560℃/ 12 h	4.2	3.9±1.5	70.9

Table S2. Comparison of Polarization current at 0.9V, ECSA and SA of Pt_3Co_2-560℃-4h, Pt_3Cu_2-600℃-4h ,Pt_3Ni_2 -550℃-8h and Pt_3Fe_2-400℃-4h catalysts.

Catalyst	Calcination program	Polarization current at 0.9V (mA cm^{-2})	ECSA (m^2 g^{-1})	SA (A mg^{-1})
Pt_3Co_2	560 ℃/ 4h	3.26	93.8	0.496
Pt_3Cu_2	600 ℃/4h	2.94	102	0.378
Pt_3Ni_2	550 ℃/8h	2.97	70.8	0.575
Pt_3Fe_2	400 ℃/4h	2.39	55.2	0.466
